# Evolutionary Computing for the Radiative–Convective Heat Transfer of a Wetted Wavy Fin Using a Genetic Algorithm-Based Neural Network

**DOI:** 10.3390/biomimetics8080574

**Published:** 2023-12-01

**Authors:** B. S. Poornima, Ioannis E. Sarris, K. Chandan, K.V. Nagaraja, R. S. Varun Kumar, Samia Ben Ahmed

**Affiliations:** 1 Department of Mathematics, Amrita School of Engineering, Amrita Vishwa Vidyapeetham, Bengaluru 560035, Karnataka, India; bl.sc.r4mat21002@bl.students.amrita.edu (B.S.P.); bl.sc.r4mat21006@bl.students.amrita.edu (K.C.); kv_nagaraja@blr.amrita.edu (K.V.N.); rs_varun@blr.amrita.edu (R.S.V.K.); 2 Department of Mechanical Engineering, University of West Attica, 12244 Athens, Greece; 3 Department of Chemistry, College of Sciences, King Khalid University, Abha P.O. Box 9004, Saudi Arabia; sbahmad@kku.edu.sa

**Keywords:** heat transfer, wet fin, wavy fin, artificial neural network, genetic algorithm

## Abstract

Evolutionary algorithms are a large class of optimization techniques inspired by the ideas of natural selection, and can be employed to address challenging problems. These algorithms iteratively evolve populations using crossover, which combines genetic information from two parent solutions, and mutation, which adds random changes. This iterative process tends to produce effective solutions. Inspired by this, the current study presents the results of thermal variation on the surface of a wetted wavy fin using a genetic algorithm in the context of parameter estimation for artificial neural network models. The physical features of convective and radiative heat transfer during wet surface conditions are also considered to develop the model. The highly nonlinear governing ordinary differential equation of the proposed fin problem is transmuted into a dimensionless equation. The graphical outcomes of the aspects of the thermal profile are demonstrated for specific non-dimensional variables. The primary observation of the current study is a decrease in temperature profile with a rise in wet parameters and convective-conductive parameters. The implemented genetic algorithm offers a powerful optimization technique that can effectively tune the parameters of the artificial neural network, leading to an enhanced predictive accuracy and convergence with the numerically obtained solution.

## 1. Introduction

Heat transfer is a mechanical engineering discipline that generates, utilizes, converts, and exchanges thermal energy (heat) through physical structures. Many thermal management or heat transmission problems have emerged in various industries due to technological advancements, including the cooling of high-power electronic devices [1,2], thermal management in engines [3,4], cooling of nuclear reactors [5,6,7], biomimetic cylinders [8], and biomimetic honeycomb fractal heat exchangers [9]. However, the design and manufacture of electrical gadgets and solar energy converters encounter a severe obstacle in the capacity to expel or absorb heat in constrained environments. The increasing requirements concerning such equipment for heat absorption and distribution have been addressed in several investigations [10,11,12,13,14]. Heat transmission enhancement strategies are critical for energy conservation and the use of appropriate energy sources. Effective heat transfer techniques have been used in various scientific domains, including chemical engineering, power generation, aircrafts, and refrigerants. The following describes the procedure for strengthening the efficiency of heat transfer systems. Two primary methods have been suggested to increase heat transfer in various contexts: passive approaches and active procedures. Active techniques such as mechanical mixing, rotation, vibration, and the incorporation of an external electrostatic or magnetic field are effectively employed to accelerate heat and mass transfer. This mechanism requires exterior force assistance to operate more quickly and efficiently. External effects may be provided to the system via a heated surface or the flow of fluids. However, in small spaces, external energy input is expensive and difficult. Conversely, passive methods can increase heat transmission by modifying the properties of the fluid and surface roughness, and mounting the objects to increase surface area. Among passive approaches, the use of nanofluids and surface modification are widely utilized and particularly effective at improving heat transmission in various engineering systems. According to many investigations, replacing conventional air cooling systems with liquid cooling systems is a viable way to meet the growing need for high heat flux expulsion while preventing temperature variations and wall overheating. Investigators have made more effort to increase the capacity of traditional fluids in heat transmission applications. In this regard, nanofluids that are finely dissipated metal or metal-oxide nanomaterials in a base liquid exhibit excellent thermal conductive properties. Moreover, the resultant nanofluid has substantially higher thermal characteristics than the base fluid. Nanofluids are used as coolants in nuclear reactors, thermal energy storage, automobile transmissions, solar power generation, electronic cooling systems, solar water heating systems, and radiators. The appealing outcomes that nanofluids yield in modern science and technology have inspired investigators to consider the thermophysical properties of nanofluids [15,16,17,18]. Many studies have been reported in the last few decades on heat transmission and nonliquid flow attributes while considering the various nanomaterials and geometries. Lu et al. [19] considered a nanoliquid containing carbon nanotubes to examine its heat transmission and flow behavior past a thin film. Turkyilmazoglu [20] probed the heat transfer rate of nanoliquid flow in a concentric annulus and concluded that the rate of heat transfer augments with the volume fraction of the nanoliquid. His study included different kinds of nanoparticles, namely Copper (Cu), Copper oxide (CuO), Silver (Ag), Alumina (Al_2_O_3_), and Titanium Oxide (TiO_2_). The effect of a chemical reaction in a stream of water-based nanofluid containing Ag was explored by Suleman et al. [21]. Haq et al. [22] discussed the thermal radiative heat transmission and flow of a nanoliquid (Cu-Al_2_O_3_–water) past an exponentially stretchy sheet. The chemical reactive flow of a nanoliquid containing CuO-Al_2_O_3_ particles was examined by Ramzan et al. [23]. The impact of heat sink/source in the stream of a titania–ethylene–glycol-based nanofluid over a stretchable cylinder was scrutinized by Alsulami et al. [24]. Alharbi et al. [25] elucidated the magnetohydrodynamic flow of a nanofluid past an elastic stretching surface. The significance of a nanoliquid (Ag-Al_2_O_3_-TiO_2_–water) in the mass and heat transference mechanism of a ternary nanoliquid was examined by Nagaraja et al. [26], with the consideration of thermal radiation. Adnan et al. [27] explained the thermal enhancement in a Cu–kerosene oil-based nanofluid stream across plates with the impact of radiation and magnetic force.

Heat transfer from various systems, such as electronic components, bio-medical functions, vehicle radiators, and heat sinks is significant. However, traditional heat transfer strategies are inefficient due to the small size, shape, and weight of the various systems. The advancement of technology necessitated the development of efficient heat transmission systems. Operating compact and smaller electronic devices that generate an exceedingly large quantity of heat, and fail to provide sufficient surface area for transferring this heat, demands the requirement for a highly effective cooling strategy. Thus, extended surfaces or fins of different shapes are used to increase surface area and thereby improve the rate of heat transmission. Fins are frequently employed in many thermal engineering applications to accelerate the rate of heat transfer from heated surfaces. Fins are useful in removing heat from other electrical components, such as computer CPUs, heat evaporators, compressors, and internal combustion engines [28,29,30,31,32]. Also, wet extended surfaces are widely implemented in refrigeration and air conditioning, where heat transfer necessitates simultaneously chilling and dehumidifying humid air at room temperature. The surface of the fin becomes wet when the actual temperature on the fin surface drops below the dew point temperature of the ambient air. In view of this context, Kundu and Lee [33] studied the heat transmission mechanism in rectangular, triangular, convex, and exponential fins by considering wetted conditions. Turkyilmazoglu [34] explored the mass and heat transmission in a fully wetted permeable fin. Das and Kundu [35] discussed the consequence of wetness on the convective heat transmission of rectangular and concave profiled fins. Hazarika et al. [36] investigated the mass and heat dissipation enhancement of a fin under wet conditions. Gamaoun et al. [37] described the mechanism of heat dissipation in a rectangular fin wetted by a zinc oxide-Society of Automotive Engineers 50 nanolubricant. Abdulrahman et al. [38] considered a nanoliquid to analyze the heat transmission in a wetted exponential fin with radiation impact.

Geometrical modifications are one of the primary methods used to increase heat transfer rates under various problem conditions. The performance of the heat transfer rate of equipment may be improved by using this simpler and most affordable technique. In this regard, a wavy-designed system is the most important model for analyzing heat and mass transmission efficiency. This kind of design is also used in cooling towers, microchips, and heat exchangers. On the other hand, compact heat exchangers with wavy fins are utilized in thermal equipment for construction, agricultural, and industrial purposes. Optimizing the air side fin arrangement is the most effective way to improve the performance of fin-and-tube heat exchangers. Employing wavy fins is the conventional method for increasing air side heat transfer. Several experimental studies have examined the efficiency of wavy fin-and-tube heat exchangers for air side heat transfer. Wavy fins have become a preferred alternative to flat fins for heat pump air conditioners. Xiao et al. [39] discussed the heat transfer enhancement in a wavy-finned flat tube bundle using water spay cooling. Wen et al. [40] studied the performance of wavy fins in plate-fin heat exchangers with the consideration of a fluid–structure interaction. Chu et al. [41] explained the air side functioning of fin-and-tube heat exchangers with sinusoidal wavy fin geometry. The study also experimentally compared heat transfer performance in round and oval tube configurations. Zhang et al. [42] studied the heat transfer of a herringbone wavy fin, applicable in the analysis of heat exchangers. Erdinc [43] examined heat transfer in circular wavy fin-and-tube heat exchangers and elliptical fin-and-tube heat exchangers.

Metaheuristic algorithms have developed effective methods for handling complicated real-world problems in various disciplines. The interaction of intensification and diversity, inspired by natural and physical processes, is vital to their problem-solving abilities [44]. These algorithms use a natural selection-like process in which viable solutions evolve over numerous generations, adapting and improving through selection, crossover, and mutation operations. Genetic algorithms (GAs) investigate a solution space by maintaining a population of potential solutions, encouraging diversity, and gradually converging on optimal or near-optimal solutions. Also, biological evolution principles inspire the GA, one of the most well-known metaheuristic algorithms [45]. GAs have been shown to be quite effective in handling a wide range of optimization and search difficulties. Improving energy efficiency in smart cities is a significant requirement; thus, Le et al. [46] proposed a unique GA-ANN model to analyze the heat load of buildings. In this work, the GA-ANN outperforms other techniques in terms of performance. These models can anticipate characteristics that are important in optimizing building design for energy efficiency and can be integrated into smart homes and municipal planning. Albadr et al. [47] proposed a GA-based natural selection technique to improve exploitation and exploration control. The proposed method surpasses the traditional GA and other optimization approaches in various tests, providing a better balance of exploration and exploitation through chromosomal selection refinement and introducing a mean-based evaluation. In the developing field of wind power, Zhang et al. [48] studied the improvement of wind speed prediction accuracy for reliable grid operation and energy delivery. The model improves prediction accuracy by applying hierarchical clustering, and optimizing artificial neural networks with evolutionary algorithms. Hamdia et al. [49] present a robust optimization strategy for machine learning models that uses evolutionary algorithms to optimize the architecture and feature configurations of the ANN model. This study was conducted in computational material design and validated the approach by boosting prediction accuracy for fracture energy in polymer–nanoparticle composites. The optimized ANN outperforms traditional models, with fewer generations in the evolutionary process. Let et al. [50] investigated the bed expansion in a binary particle combination within Newtonian liquid in circular columns. The findings reveal that bed expansion increases with increasing liquid velocity and decreases with increasing particle diameter. The combination of GA and neural networks improves the accuracy of bed height forecasts, providing valuable tools for future research in this area. Sharifi et al. [51] presented an ANN that outperforms semi-empirical correlations in estimating heat transfer rates and friction coefficients for heat exchangers with coiled wire within input variable ranges. The application of the GA in conjunction with ANNs in the study determines the ideal spiral wire structure for the highest overall efficiency enhancement in heat exchangers, offering valuable insights for heat exchanger design and optimization. Wen et al. [52] inspected the ZnO–water nanofluid’s thermal and flow properties in multiport mini-channels. Heat transfer performance is predicted in this study using an ANN-based genetic approach. Cui et al. [53] examined the impact of a metal foam–fin hybrid structure and inclination angle on the heat transfer performance of a phase change material. The implemented GA-ANN model effectively predicts liquid fractions and Nusselt numbers during the phase change.

The typical approach for enhancing air side heat transfer involves using a wavy extended surface. In the aforementioned literature, it is noted that several researchers investigated heat transmission in a wavy profiled fin. However, heat transport in a wet wavy fin with temperature-dependent thermal conductivity has not yet been studied. On the other hand, optimization strategies, which are a subset of intelligence techniques, can be used for analyzing mathematical models. A genetic algorithm is a prominent evolutionary method for achieving this objective. Thus, the current study focuses on analyzing heat transmission of a wet wavy fin with variable thermal conductivity using an optimization algorithm. Also, the heat transport behavior of the wet wavy fin is studied by employing a genetic algorithm-enhanced neural network (GA-ENN). Further, the consequence of different constraints on the thermal profile of the wavy fin is exhibited with the help of graphs. Numerous investigations on the thermal characteristics of various fin designs have been reported in the literature. For a more comprehensive understanding, Table 1 provides recent studies on fin structure while considering the wet conditions.

It is evident from Table 1 that the thermal features of wavy fins have been given less importance. The pioneering of the present research over previous investigations can be described as follows:

- Presenting a mathematical model for thermal transfer in a wetted wavy fin with convection and radiation mechanisms to inspect the heat transport features of the fin.
- Analyzing the impact of radiation and surface wetness on the thermal performance of the wavy fin.
- An artificial intelligence-based genetic algorithm is used to examine the heat transfer rate of the wavy fin. Implementing this sophisticated optimization method and better computing technology has enabled an estimation of the thermal characteristics of the wetted wavy fin.
- Comparing the thermal variation attributes of dry and wet wavy fins under the influence of convection and radiation.

The current investigation has been organized according to the following: Section 2 describes the mathematical model of the problem, including an explanation of the physical illustration of the wavy fin, the fundamental governing equations, the appropriate boundary conditions, and the applied dimensionless variables. Section 3 discusses the general procedures of genetic algorithm-enhanced neural networks. Tables and graphs are used to explore the results, which are reported in Section 4. Section 5 contains the conclusions, which provide an overview of the major results.

## 2. Formulation of the Problem

**Figure 1:**
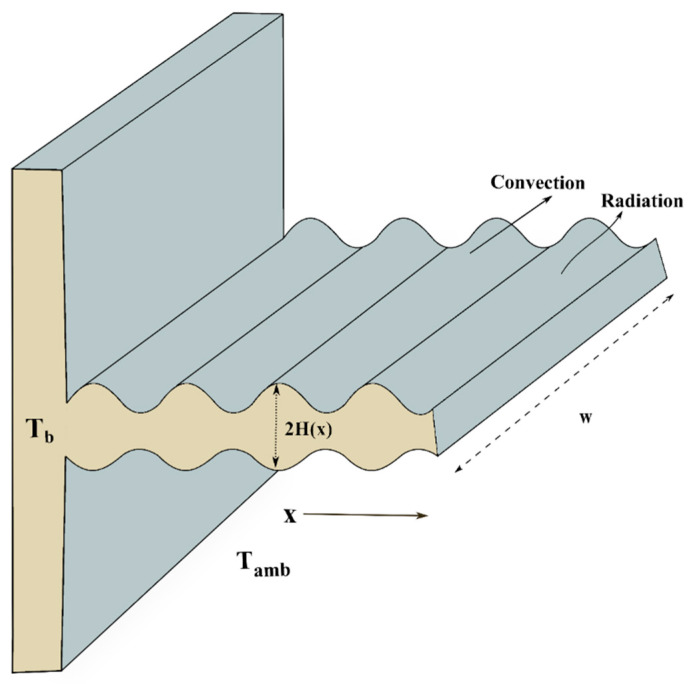
Pictorial design of a wavy fin.

Heat transmission in a wavy profiled rectangular fin with temperature-dependent thermal conductivity is considered in the current study. At ambient temperature $T_{amb}$, the wavy profiled fin is exposed to a convective environment and $T_{b}$ is assumed as the base temperature on the surface of the wetted wavy fin (SWWF). The height and width of the wavy fin are assumed to be 2H and w. The considered rectangular fin is wavy along the *x*-axis. Figure 1 illustrates the physical depiction of SWWF. The following governing equation provides the mathematical representation of the wavy fin problem (Kundu and Yook [61], and Khaled [64]).

(1) $$\begin{array}{l} \frac{d}{dx}\left[k^{*}\left(T\right)\frac{dT}{dx}\right]=\left[\frac{dA_{SF}}{dx}\right]\frac{h^{*}\left(T\right)}{A_{CS}}\left(T-T_{amb}\right)-\left[\frac{k^{*}\left(T\right)}{A_{CS}}\frac{dA_{CS}}{dx}\right]\frac{dT}{dx}\\ +\left[\frac{dA_{SF}}{dx}\right]\frac{\sigma \varepsilon^{*}}{A_{CS}}\left(T^{4}-T_{amb}^{4}\right)+\left[\frac{dA_{SF}}{dx}\right]\frac{h_{D}i_{fg}\left(\omega -\omega_{amb}\right)}{A_{CS}} \end{array}$$

where, $k^{*}\left(T\right)$ is the thermal conductivity of the wavy fin,

(2) $$k^{*}\left(T\right)=k_{amb}\left[1+\tilde{\kappa }\left(T-T_{amb}\right)\right]$$

According to the Chilton–Colburn analogy, heat and mass transport have the following relationship,

(3) $$h^{*}(T)=h_{b}{\left(\frac{T-T_{amb}}{T_{b}-T_{amb}}\right)}^{m}=h_{D}c_{p}Le^{\frac{2}{3}}$$

Substituting the Equations (2) and (3) in Equation (1) yields,

(4) $$\begin{array}{l} \frac{d}{dx}\left[k_{amb}\left[1+\tilde{\kappa }\left(T-T_{amb}\right)\right]\frac{dT}{dx}\right]=\left[\frac{dA_{SF}}{dx}\right]\frac{h_{b}}{A_{CS}}\frac{{\left(T-T_{amb}\right)}^{m+1}}{{\left(T_{b}-T_{amb}\right)}^{m}}\\ -\left[\frac{k_{amb}\left[1+\tilde{\kappa }\left(T-T_{amb}\right)\right]}{A_{CS}}\frac{dA_{CS}}{dx}\right]\frac{dT}{dx}+\left[\frac{dA_{SF}}{dx}\right]\frac{\sigma \varepsilon^{*}}{A_{CS}}\left(T^{4}-T_{amb}^{4}\right)\\ +\frac{1}{A_{CS}}\left[\frac{dA_{SF}}{dx}\right]\frac{h_{b}i_{fg}b_{1}}{c_{p}Le^{\frac{2}{3}}}\frac{{\left(T-T_{amb}\right)}^{m+1}}{{\left(T_{b}-T_{amb}\right)}^{m}} \end{array}$$

The cross-sectional area is represented as (Khaled [64])

(5) $$A_{CS}=2H_{0}\int_{0}^{w}\left\{1+\delta \sin\left[2\pi n\left(\frac{x}{L}\right)+\varphi \right]\right\}dz$$

and

(6) $$H=H_{0}\left\{1+\delta \sin\left[2\pi n\left(\frac{x}{L}\right)+\varphi \right]\right\}$$

Also, the fin surface area is provided by

(7) $$A_{SF}=2wL_{ta}$$

where, $L_{ta}=\int_{0}^{L}\sqrt{1+{\left(\frac{dH}{dx}\right)}^{2}}dx$.

The heat equation of the considered fin problem can be solved by using appropriate boundary conditions.

$$x=0:T=T_{b},$$

(8) $$x=L:\frac{dT}{dx}=0$$

Suitable dimensionless variables for the balanced energy equation are given below,

$$\Theta =\frac{T}{T_{b}},\;\Theta_{amb}=\frac{T_{amb}}{T_{b}},\;X=\frac{x}{L},\;\beta =\tilde{\kappa }T_{b},\;Nc=\frac{h_{b}L^{2}}{k_{amb}H_{0}},\;a_{RL}=\frac{H_{0}}{L},$$

(9) $$Nr=\frac{\sigma \varepsilon^{*}L^{2}T_{b}^{3}}{k_{amb}H_{0}},\;\left(\omega -\omega_{amb}\right)=b_{1}\left(T-T_{amb}\right),\;\chi =\frac{h_{b}i_{fg}b_{1}L^{2}}{H_{0}c_{p}Le^{\frac{2}{3}}k_{amb}}$$

After utilizing these dimensionless variables, the obtained non-dimensional temperature equation is specified below,

(10) $$\begin{array}{l} \frac{d}{dX}\left[\left(1+\beta \left(\Theta -\Theta_{amb}\right)\right)\frac{d\Theta }{dX}\right]+\left(1+\beta \left(\Theta -\Theta_{amb}\right)\right)\left[\frac{2\pi \delta n\cos\left(2\pi nX+\varphi \right)}{1+\delta \sin\left(2\pi nX+\varphi \right)}\right]\frac{d\Theta }{dX}\\ -Nc\left[\frac{\sqrt{1+4{\left(\pi a_{RL}\delta n\right)}^{2}\cos^{2}\left(2\pi nX+\varphi \right)}}{1+\delta \sin\left(2\pi nX\right)}\right]{\frac{\left(\Theta -\Theta_{amb}\right)}{{\left(1-\Theta_{amb}\right)}^{m}}}^{m+1}\\ -Nr\left[\frac{\sqrt{1+4{\left(\pi a_{RL}\delta n\right)}^{2}\cos^{2}\left(2\pi nX+\varphi \right)}}{1+\delta \sin\left(2\pi nX\right)}\right]\left(\Theta^{4}-\Theta_{amb}^{4}\right)\\ -\chi \left[\frac{\sqrt{1+4{\left(\pi a_{RL}\delta n\right)}^{2}\cos^{2}\left(2\pi nX+\varphi \right)}}{1+\delta \sin\left(2\pi nX\right)}\right]{\frac{\left(\Theta -\Theta_{amb}\right)}{{\left(1-\Theta_{amb}\right)}^{m}}}^{m+1}=0 \end{array}$$

The boundary condition is reduced using Equation (9) as,

(11) $$\begin{array}{l} X=0:\Theta =1,\\ X=1:\Theta^{\prime }=0. \end{array}$$

The dimensionless expression for the heat transfer rate Q is mathematically expressed as:

(12) $$Q=-a_{RL}\left[1+\beta \left(\Theta \left(0\right)-\Theta_{amb}\right)\right]{\left.\Theta^{\prime }\right|}_{X=0}$$

## 3. Genetic Algorithm-Enhanced Neural Network (GA-ENN)

**Figure 2:**
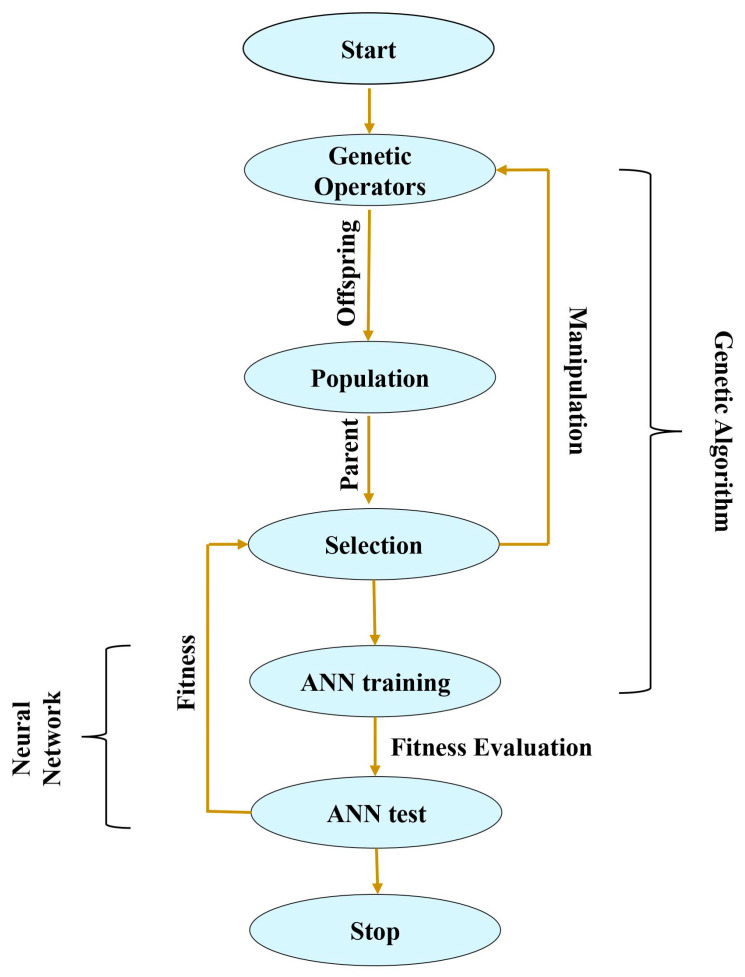
The GA-ANN model workflow.

Artificial Neural Networks (ANNs) have emerged as a key technique in machine learning and artificial intelligence. These models imitate the linked network of neurons in the human brain, and have widespread applications. These frameworks make neural network design and training easier, making them a popular choice for machine learning projects. ANNs are used in scientific research in drug development, climate modelling, and genomics. They make pattern recognition in complex datasets easier, allowing researchers to discover insights that were previously difficult to extract. ANNs aid in stock price prediction, fraud detection, and algorithmic trading in the finance sector by analyzing massive amounts of financial data with great accuracy. Their capacity to spot complex patterns in data is a tremendous advantage. ANNs are also employed in autonomous cars for obstacle detection and path-planning tasks. They help in illness diagnosis and medical imaging interpretation in healthcare, increasing the precision of medical experts. They analyze user behavior and preferences to give personalized content and product recommendations. In machine learning, optimizing model parameters is vital for obtaining better performance. The understanding of the complex interplay between the Genetic Algorithm (GA) and parameter estimation in the Artificial neural network (ANN) model can be advantageous for forecasting many problems [46,48]. The ANNs are adaptable instruments for complicated data modelling, requiring a careful selection of parameters to reach their full potential. The GA emulates the natural selection and evolution process to identify optimal solutions, which comprises key elements such as encoding, selection, genetic operators (crossover and mutation), and fitness evaluation. The GA iteratively converges towards solutions that satisfy the given objectives in ANN parameter estimation. It plays a pivotal role in determining the optimal architecture combination that minimizes the error between predicted and actual outcomes. Figure 2 presents a flowchart for integrating the GA with ANN parameter estimation, unfolding as a multi-faceted process. It commences with encoding model parameters into chromosomes, which serve as the genetic material for evolution. A diverse population of potential solutions is then generated, representing various parameter configurations. The fitness evaluation comes first, as the ANN model with specific parameter sets is rigorously assessed through a well-defined fitness function, the mean squared error (MSE). The selection mechanism favors the solutions with higher fitness, ensuring a promising parameter configuration persists. Through crossover and mutation operations, the concept of genetic diversity is introduced. This strikes a balance between exploration of new configurations and exploitation. The termination criteria is often defined by the number of generations or convergence that signify the culmination of the GA iteration, presenting the most optimal parameter set for the given task. The performance indicators are helpful in the justification of efficiency for the proposed model with maximum error (ME), and Equations (13)–(15) present the mean absolute error (MAE), coefficient of determination ($R^{2}$), and MSE. The solving procedure of the GA-ANN is presented in the form of the algorithm as shown in Algorithm 1:
**Algorithm 1** Solving procedure for the proposed problem using GA-ANN**Step 1:** Start.**Step 2:** Split the whole dataset into training and testing subsets for data preparation.**Step 3:** Initialization of the population of the neural network configuration using binary encoding.**Step 4:** Building a network architecture and hyper-parameters based on individuals and training on the provided data set.**Step 5:** Evaluating the performance of the model using the mean square error fitness function on the testing data.**Step 6:** Create a population that can adapt to various combinations of architecture and hyper-parameter settings.**Step 7:** Performing an iterative loop for the following genetic operation:
**a.** **Selection:** Choose the parents of the next generation via tournament selection.**b.** **Crossover:** Use crossover to combine the architecture and hyper-parameters of the parent pairs.**c.** **Mutation:** Make random alterations to the architecture and hyper-parameters of the offspring.**d.** **Evaluation:** Determine the fitness of the offspring.**e.** **Survival selection:** Based on fitness, select the next generation.**f.** **Increment generation:** Increase the generation counter.**Step 8:** The individual with the lowest MSE is chosen as the best solution in the final population.**Step 9:** Employ the optimized architecture and hyper-parameters to build and train a neural network for regression on the complete dataset.**Step 10:** Stop.

(13) $$MAE=\frac{1}{j}\sum_{i=1}^{j}\left|y_{act}-y_{pred}\right|$$

(14) $$R^{2}=1-\frac{\sum_{i=1}^{j}{\left(y_{act}-y_{pred}\right)}^{2}}{\sum_{i=1}^{j}{\left(y_{act}-y_{mean}\right)}^{2}}$$

(15) $$MSE=\frac{1}{j}\sum_{i=1}^{j}{\left(y_{act}-y_{pred}\right)}^{2}$$

(16) $$Gelu=\frac{1}{2}\zeta_{input}\left[1+\tanh\left(\sqrt{\frac{2}{\pi }\left(\zeta_{input}+0.044715\zeta_{input}^{3}\right)}\right)\right]$$

## 4. Results and Discussion

The thermal variation and heat transfer of a wetted wavy fin are explored under the impact of convection and radiation. The heat transmission equation of the wavy profiled wet fin is considered in Equation (1) and this equation is non-dimensionalized with the help of dimensionless constraints. The Runge–Kutta–Fehlberg’s fourth–fifth (RKF-45) approach is utilized to solve a mathematical model of ODE involving the surface emissivity, convection, and wet conditions of the wavy fin. The applied GA-ANN methodology is trained in Jupyter Notebook version: 6.5.5 and is developed using Python frameworks such as TensorFlow v2.14.0 and Keras version 2.14.0. The influence of various parameters, namely Nc (convection–conduction parameter), β (thermal conductivity parameter), and Nr (radiation–conduction parameter) on the temperature profile of the wavy fin is portrayed with the help of graphs. In summary, this sector has been divided into two sections. The first segment shows the temperature distribution for the wavy wet and dry fins as a consequence of their thermal parameters. The results for the GA-ANN heat transfer rate Q are included in the other segment.

### 4.1. Impact of Thermal Parameters on the Temperature Profile

**Figure 3:**
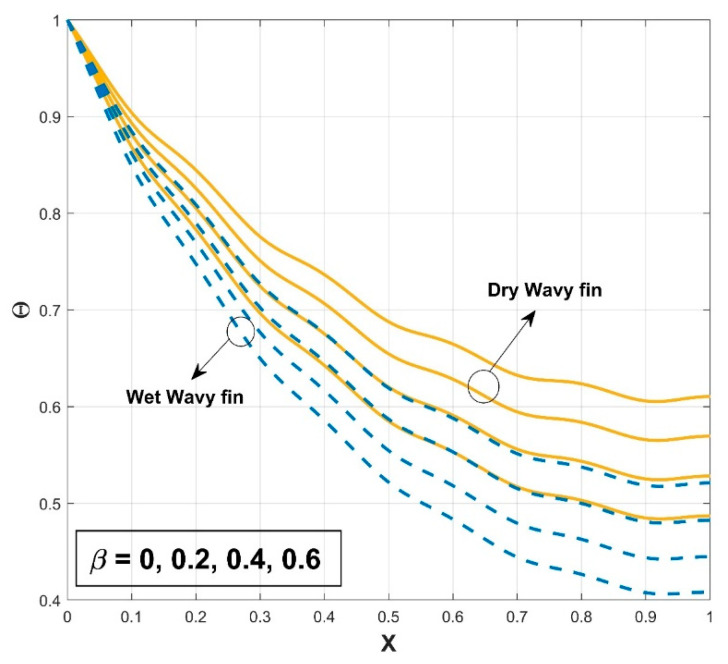
Variation in Θ against β for SWWF and SDWF.

**Figure 4:**
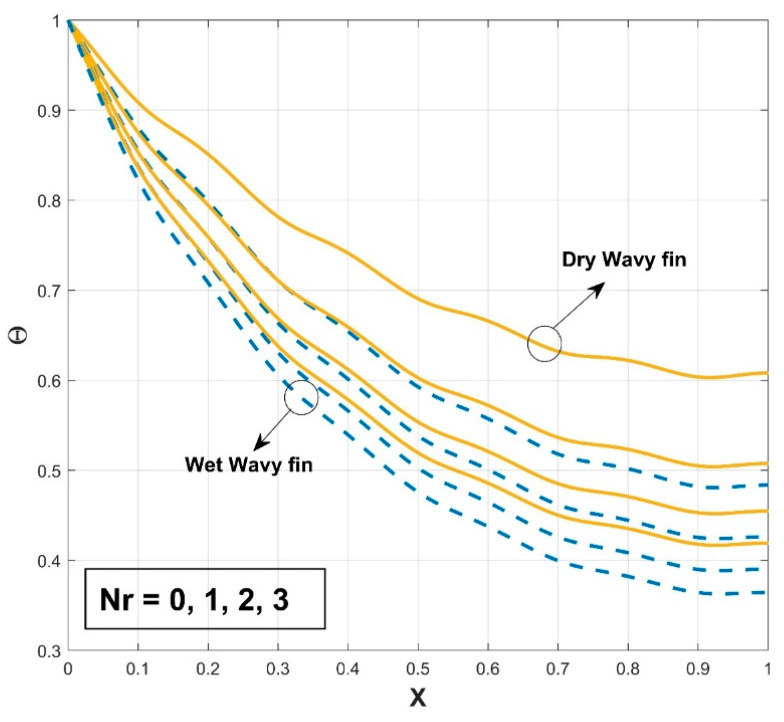
Variation in Θ against Nr for SWWF and SDWF.

**Figure 5:**
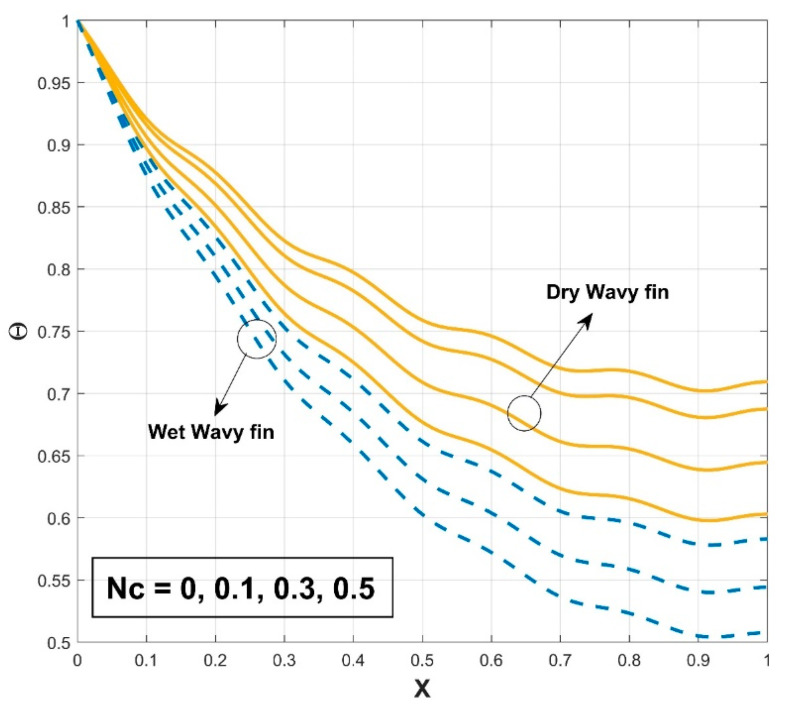
Variation in Θ against Nc for SWWF and SDWF.

**Figure 6:**
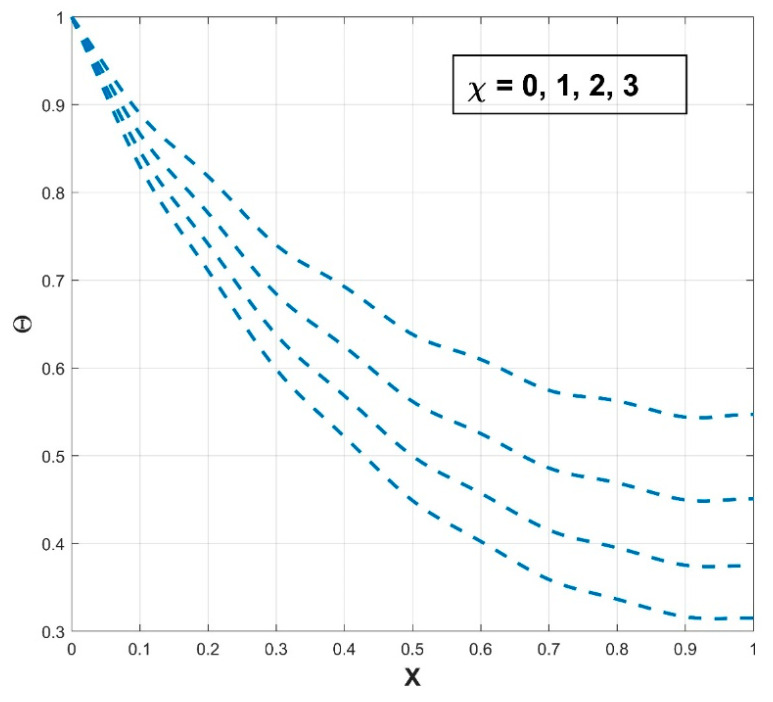
Variation in Θ against χ for SWWF.

Figure 3 presents the influence of the thermal conductivity parameter β on the thermal profile of the SWWF. The provided graph shows that the thermal profile exhibits an increasing trend with a gradual escalation in the β scales. Also, the significance of surface wetness is graphically indicated by comparing it to the dry condition. The temperature curve of the fin for wet conditions is comparatively lower than in dry conditions, indicating the decrease of the temperature distribution in the fin with wet conditions. It can be concluded that the heat transfer rate is higher for SWWF than for the surface dry wavy fin (SDWF). Enhancing the thermal conductivity gradient raises the temperature within the fin and promotes heat transfer from the fin base. The temperature distribution across the fin becomes better dispersed and the fin becomes more conductive with the increase of β. The radiation impact on the thermal variance of the SWWF is discussed in Figure 4, along with the associated dimensionless radiative–conductive variable. Improvement in the thermal variation is exhibited with a decrease in the radiative–conductive variable for the considered SWWF and SDWF. As radiation strength increases, the efficiency of the fin in transmitting heat to the surrounding fluid is strengthened, which is revealed by the decreasing temperature. The findings suggest that radiation-induced thermal energy exchanges are raising the heat transfer rate of the fin. The surface characteristics of the fin and the temperature variance between it and the surrounding air determine the strength of the radiation flux. A similar fashion in the thermal profile is observed as a consequence of the convective–conductive variable, as revealed in Figure 5. In particular, a decrease in the values of this variable stimulates the temperature distribution in the wavy fin for both the SWWF and SDWF. The convective heat transfer coefficient and Nc are directly correlated, as shown in Equation (9). Thus as Nc grows, the cooling rate also improves and the temperature of the fin falls as a consequence. Figure 6 expounds the wetness effect on the thermal performance of the fin via a dimensionless wet parameter. An increase in the magnitude of this parameter decreases the thermal variation in the wetted wavy fin, thereby promoting a higher rate of heat transmission in the fin. The moist condition increases the fin’s cooling effect by removing excess heat from the fin’s surface.

### 4.2. Impact of the GA-ANN on Heat Transfer Rate

The application of the GA in estimating parameters within an ANN model gives an appealing avenue for optimizing complex structures and improving predictive performance. The current work designs an architecture determined by the GA, which demonstrates the astonishing ability of the GA to identify optimal model hyper-parameter sets. The GA works based on the evolutionary principle, imitating the process of natural selection to iteratively refine solutions and achieve optimal results. By navigating large and high-dimensional search spaces in the context of an ANN model parameter estimation, the GA-driven optimization process involves several key stages. To begin, the ANN hyper-parameters, which include a hidden layer architecture, activation function, and batch size are encoded into genetic representations, resulting in the first population of potential solutions. The fitness of these solutions is then assessed by the GA using performance metrics such as MSE, which quantifies the difference between projected and actual outputs. This evaluation directs the selection of potential parameter combinations of the algorithm, allowing it to prioritize and generate solutions with higher fitness values. The GA uses genetic operators such as crossover and mutation to change and refine the encoded parameter sets throughout generations. The combining of genetic information from two parent solutions results in offspring who inherit qualities from both. Mutation introduces controlled modifications into the genetic process, allowing for the investigation of novel parameter configurations. The capacity of the GA to achieve a balance between exploration and exploitation is critical to its success in estimating ANN model parameters. The algorithm investigates various parameter combinations while focusing on accurate outcomes. This adaptability is especially useful in the context of complicated neural network topologies. It enables the GA to traverse extensive search spaces and find optimal solutions that would be difficult to assess using manual or gradient-based approaches.

**Figure 7:**
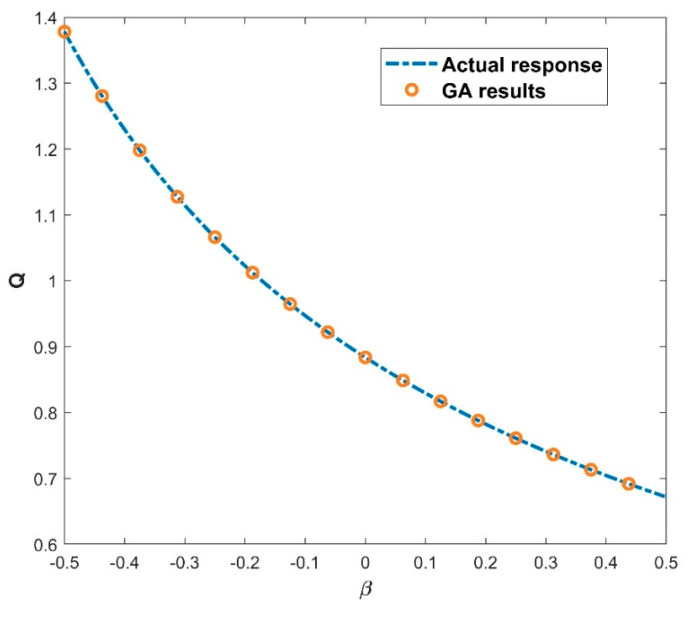
Variation in Q against β for the GA results.

**Figure 8:**
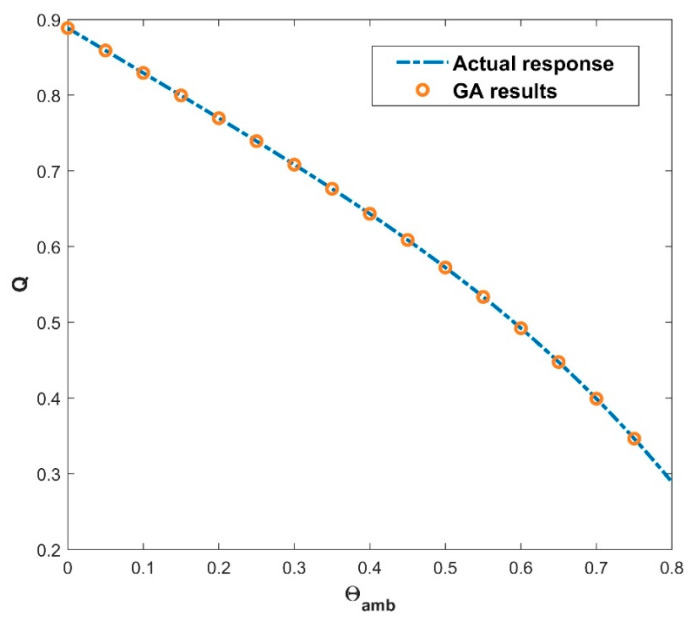
Variation in Q against $\Theta_{amb}$ for the GA results.

**Figure 9:**
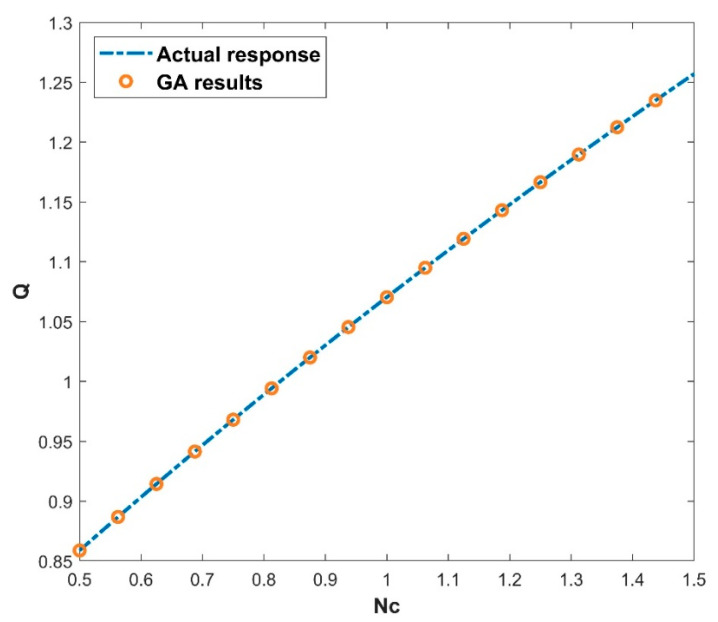
Variation in Q against Nc for the GA results.

**Figure 10:**
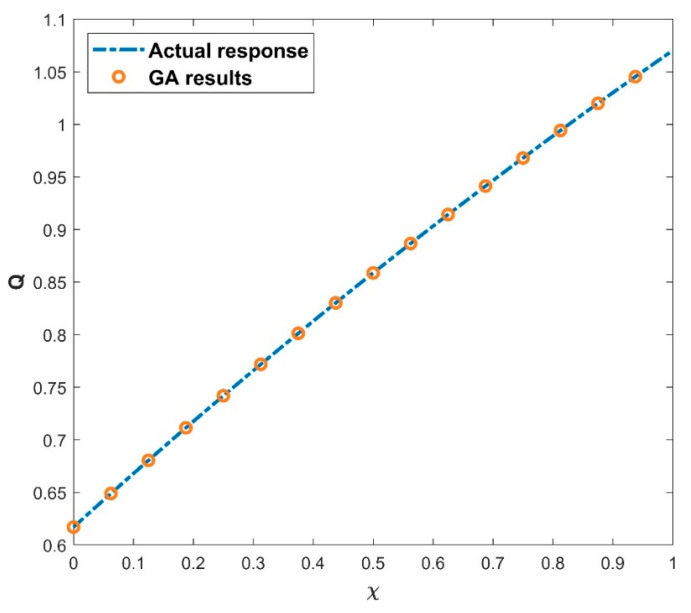
Variation in Q against χ for GA results.

**Figure 11:**
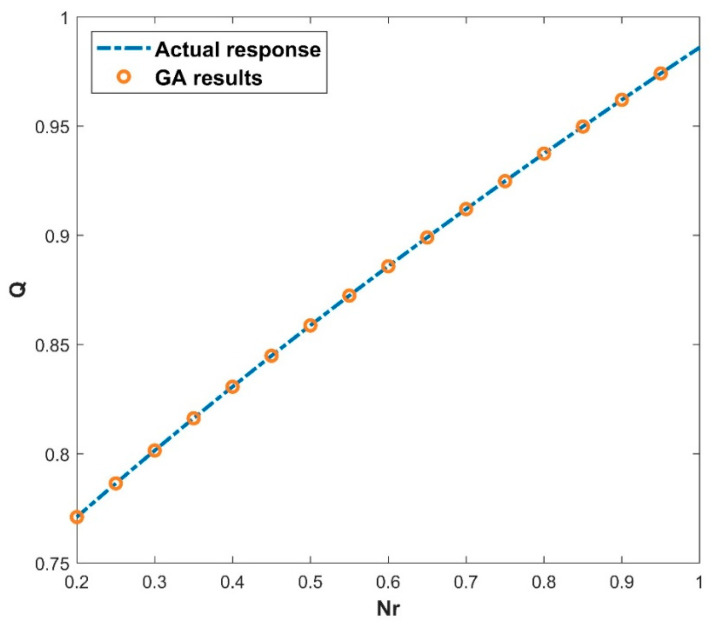
Variation in Q against Nr for the GA results.

**Figure 12:**
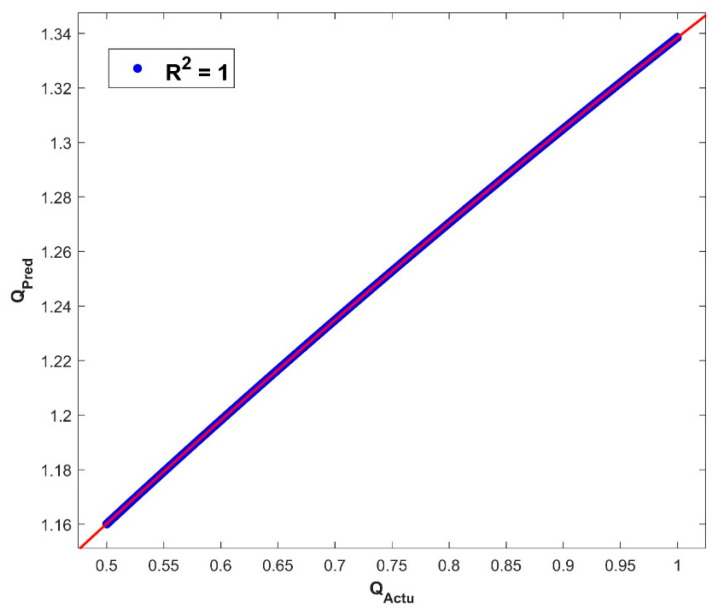
Comparison of actual and predicted Q results.

**Figure 13:**
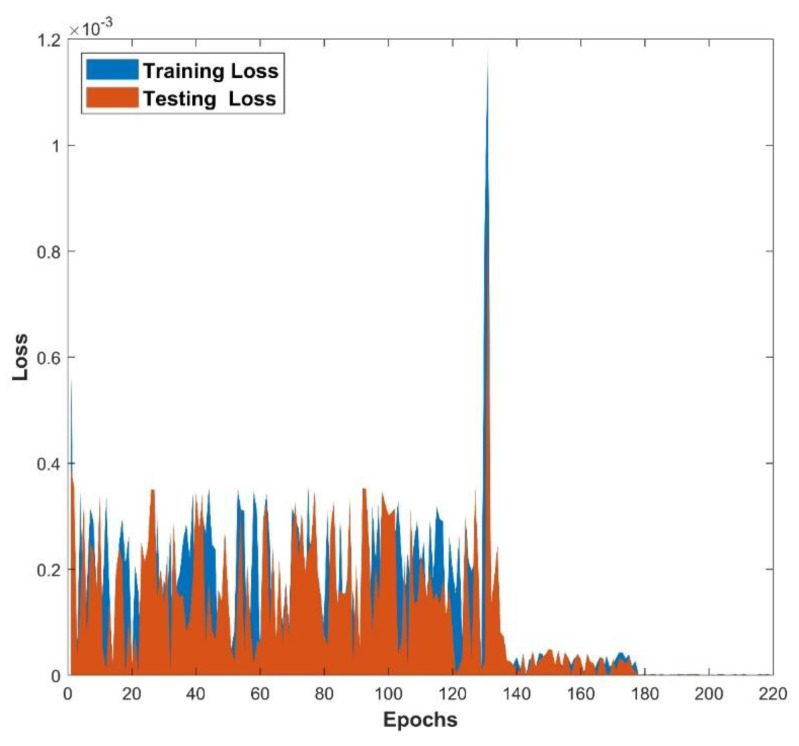
Training and testing results of the GA-ANN.

The architecture of 64, 48, and 32 neurons arranged in hidden layers, together with the GELU activation function (as seen in Equation (16)) and a batch size of 27, demonstrates the GA’s prowess in unravelling the ANN model’s underlying structure. The GA’s ability to optimize these parameters across 697 epochs demonstrates its skill in navigating a large solution terrain and efficiently fine-tuning the ANN model for improved predicting accuracy. The use of the GA to find the best architecture shows its capacity to optimize ANNs for higher performance, resulting in more accurate predictions and refined model outputs. The GA-ANN model is validated and its empirical performance is depicted in a series of five interconnected graphs, each depicting the influence of the corresponding thermal variables. The variation in the values of thermal variables is taken as input data along the *x*-axis, while simultaneously exhibiting the actual response and anticipated outcomes of the heat transfer rate $\left(Q\right)$ on the *y*-axis. Figure 7 prominently displays the convergence between the actual reaction and the model’s predictions of Q, providing immediate insight into the model’s capacity to closely mimic real-world behavior. This also includes the heat transfer rate variation with the influence of the thermal conductivity parameter. Increase in the values of β declined the rate of heat transfer. Elevating the level of thermal conductivity accelerates the transfer of heat from the fin base, resulting in elevated temperatures within the fin. The heat transfer of the wavy fin has increased by 51.25% in the particular case of β from 0.5 to −0.5. In addition, Figure 8 emphasizes the precision of the model as $\Theta_{amb}$ progresses on the *x*-axis. The resulting convergence of the actual and anticipated responses of Q provides visual evidence to the usefulness of GA-ANN model. The heat transfer rate is observed to diminish with an increase in the values of $\Theta_{amb}$. As $\Theta_{amb}$ rises, the convective heat loss from the fin declines, resulting in an elevated dimensionless temperature in the fin. When considering the $\Theta_{amb}$ value from 0 to 0.8, Q decreases by 67.48% due to the convective heat loss. Moving on, Figure 9 shows excellent alignment between the model’s predictions and the actual response of Q against the improving attributes of Nc, highlighting the accuracy of the GA-ANN in producing predictions that closely match true observations. Gradual variation in the heat transport rate is caused due to the rise in Nc values. It is observed that the natural convection phenomena cause the fin surface temperature to decrease and the heat transfer rate to elevate with an upsurge in the convection parameter Nc. As the magnitude of Nc increases from 0.5 to 1.5, Q increases to 46.37%. The versatility of the GA-ANN in interpreting sophisticated data links of Q versus χ is evident in Figure 10. The line alignment in this figure shows the ability of the GA-ANN to reveal complex patterns within the input data of χ. The upshot in the values of the wet parameter causes an upsurge in the heat transfer rate. The wetness around the fin is helpful in absorbing more heat from the surface of the fin. As a result, the heat transfer rate is improved and the fin temperature is lowered. The maximum 73.48% heat transfer rate is yielded with an increase in χ from 0 to 1. Finally, Figure 11 summarizes the pictorial narrative by integrating all of the visualizations to highlight the consistent achievement of the GA-ANN model in providing predictions of Q that reliably agree with observations across varied Nr. Improvement in the Nr scales promotes the rate of heat transmission. The internal temperature of the fin decreases as radiation intensity develops, indicating that the fin efficiently transfers heat to the surrounding fluid. Based on the evidence, it can be concluded that radiation-induced temperature heat transmissions augment the heat transfer rate of the fin. In particular, the heat transfer rate is augmented by 27.88% for a rise in Nr from 0.2 to 1. The relationship between the actual data and the predicted outputs of Q, determined by both the training and test data sets, can be seen in Figure 12. The results shown here demonstrate good convergence of the outcomes. The training and testing outcomes of the implemented GA-ANN model are shown in Figure 13. This graph depicts the difference between the actual and predicted (i.e., loss error) values in the training and testing phases. The estimated loss demonstrates that the accuracy improvements of the considered thermal problem is significantly impacted by applying the GA-ANN.

Table 2 provides a performance evaluation of the GA-based ANN model, shedding insight on its prediction accuracy. Key performance indicators evaluate the training and testing phases with values of 4.51 × 10^−6^ and 4.45 × 10^−6^ for training and testing, respectively. MAE, a measure of the average difference between predicted and actual values, exhibits excellent precision. The flawlessness of the R^2^ values, indicating 1 for training and testing, demonstrates the capacity of the GA-ANN to capture data variation, demonstrating that the model accounts for 100% of the variability in the data. The MSE and AE provide additional insight into the model’s performance, signifying minor differences between the predicted and actual values, with the training MSE at 2.69 × 10^−11^ and the testing MSE at 2.64 × 10^−11^. AE values of 8.87 × 10^−6^ and 8.81 × 10^−6^ confirm the precision of the model. This table emphasizes the remarkable accuracy of a GA-based ANN model in both the training and testing stages, as well as its ability for parameter estimation and predictive precision. Several distinct advantages highlight the usefulness of the GA in the estimation of ANN parameters. These include their prowess in global optimization, which allows for effective navigation across complex and high-dimensional search environments. Their capacity to sweep across a wide range of parameter combinations while meticulously tracking down the best possible solutions equips them to address the complexities of this high-dimensional space, adeptly allowing the GA to find optimal solutions in complex environments. Furthermore, the parallelizability of the GA accelerates the optimization process, allowing for faster convergence to superior solutions. The applied GA does not require prior knowledge, making them suitable for optimizing non-continuous, noisy, and non-differentiable fitness functions.

In addition, Table 3 presents the comparison of Q versus various parameters using the GA-ANN, with intention of making more obvious judgments about the results for comparative purposes. The excellent accuracy of the Q results generated through the GA-ANN is evident from the fact that it has an error of the lowest order. This table also shows the influence of parameters β, $\Theta_{amb}$, Nc, χ, and Nr on heat transfer rate enhancement using the GA-ANN technique. Notably, decreasing the values of β consistently resulted in a higher heat transfer rate. The percentage (%) difference in heat transfer between consecutive readings is observed. When the β parameter varies from 0.1 to 0 and 0 to −0.1, the heat transfer rate increases by 7.18% and 6.54%, respectively. Similarly, decrements in $\Theta_{amb}$ values result in improved heat transfer rates by 11.12% and 12.90%. Furthermore, changing Nc from 0.6 to 0.8 resulted in a considerable increase in the heat transfer rate from 4.80% to 4.46%. Also, increasing χ from 0.1 to 0.3 resulted in a significant improvement, with the heat transfer rate increasing to 7.43% and 6.73%. When the Nr value is changed from 0.25 to 0.45, an overall increase of 7.42% in Q is observed. Table 4 compares the results of Khaled [64] against the outcomes of the ANN and GA-ANN at various Nc values by considering β=0, $\Theta_{amb}=0$, $a_{RL}=0$, χ=0, n=2, m=0, and Nr=0. This evaluation is based on absolute error percentages with further insights into the GA-ANN results. Notably, the computed results of the GA-ANN technique exhibit higher convergence with the standard ANN and the outcomes of Khaled [64]. It continuously generates decreased error percentages, suggesting a proclivity for optimizing neural network design and hyper-parameters, especially in setting a wide range of Nc values. This shows that the systematic and adaptive character of the GA-ANN, driven by its genetic algorithms, allows it to fine-tune neural network designs more successfully, resulting in increased overall performance. The majority of thermal energy applications, including power generation, air conditioning, and refrigeration, utilize wavy-shaped fins. These fins are quite popular in heat exchangers because they are inexpensive, very reliable when operating, and simple to install and maintain. Therefore, the proposed investigation contributes to determining all aspects of heat transmission in the wavy fin under wet conditions. In addition, the impact of several physical mechanisms on the thermal distribution of the wavy fin is described to explore heat and mass transference in the fin.

## 5. Conclusions

The current research focuses on examining thermal variation in the wetted wavy fin with temperature-dependent thermal conductivity, and the heat transport behavior of the wet wavy fin is explored using the GA-ANN. Additionally, the impact of various parameters on the thermal profile of the wavy fin is demonstrated with the aid of graphs. The non-dimensional energy equation of the wavy fin is numerically solved by employing the RKF-45 scheme. The major findings of the present study are listed below.

- The impact of convection on the temperature distribution of SWWF induces a higher heat dissipation rate. Convective heat loss causes heat dissipation to improve by 46.37% when the convective–conductive value varies from 0.5 to 1.5.
- The effective conductive process causes the temperature variation in the SWWF to increase, with an improvement in the thermal conductivity parameter. The heat transmission of the fin has risen by 51.25% in the case of the thermal conductivity parameter rising from 0.5 to −0.5.
- In relation to the wet parameter, the surface wetness condition of the SWWF results in a lower thermal distribution and a higher heat transfer rate. When the wet parameter increases from 0 to 1, the heat transmission rate upsurges at an average of 73.48%.
- The mechanism of heat dissipation from the fin is significantly facilitated by thermal radiation. As the radiative–conductive variable decreases, the variation in the thermal distribution within the SWWF also increases. The increase in the radiative–conductive variable from 0.2 to 1 results in a 27.88% improvement in the heat transmission rate.
- The symbiotic interaction between the GA and parameter estimation within the ANN model provides a new era of optimization possibilities.
- The estimated results of the GA-ANN technique reveal closer convergence compared to the standard ANN and the findings of previous studies.
- The GA provides a robust technique for navigating complex parameter spaces, revealing optimal configurations that improve ANN precision and convergence. This serves as a tool for computing the machine learning validation of highly nonlinear data that are numerically extracted.

The study presented in this article has drawn attention to the application of wavy fins and their thermal analysis. The development and outcomes of the investigated model have significance and can be utilized as guidance for researchers investigating the thermal assessment of various fins. The present investigation can be extended to include heat analysis in wavy fins by incorporating various sorts of artificial intelligence-based computing approaches. 

## Figures and Tables

**Table 1:** Recent investigations in the field of extended surface.

Authors	Types of Fins	Methodology	Surface Wet Condition
Hosseinzadeh et al. [54]	Trapezoidal, concave and convex parabolic fins	Akbari-Ganji’s method	✓
Gouran et al. [55]	Rectangular fin	Method of Moments and Least Square Method	✕
Ullah et al. [56]	Triangular fin	Differential transform method	✕
Adnan et al. [57]	Annular fin	Runge-Kutta Fehlberg’s fourth-fifth method	✓
Gireesha et al. [58]	Cylindrical pin fin	Finite difference method	✓
Hashemi et al. [59]	Rectangular, triangular, convex parabolic and concave parabolic fin	Iterative compact finite difference method	✓
Kumar et al. [60]	Wavy fin	Levenberg-Marquardt neural network	✕
Kundu and Yook [61]	Rectangular fin	Adomian decomposition method	✓
Kumar et al. [62]	Wavy fin	Differential evolution	✕
Din et al. [63]	Trapezoidal and exponential fins	Runge-Kutta fourth-fifth and shooting method	✕

**Table 2:** Performance evaluation of the GA-based ANN model.

Performance Indicators	Training	Testing
MAE	4.51 × 10^−6^	4.45 × 10^−6^
R^2^	1	1
MSE	2.69 × 10^−11^	2.64 × 10^−11^
AE	8.87 × 10^−6^	8.81 × 10^−6^

**Table 3:** Heat transfer values for different parameters using the GA-based ANN model.

Parameters		Q		
		RKF-45	GA-ANN	Error (%)
β	−0.1	0.94712448	0.94711942	5.34 × 10^−4^
	0	0.88364505	0.883644748	3.42 × 10^−5^
	0.1	0.82939014	0.82938141	1.05 × 10^−3^
$\Theta_{amb}$	0	0.88860663	0.88860044	6.97 × 10^−4^
	0.15	0.79963302	0.79962551	9.39 × 10^−4^
	0.30	0.7082343	0.70823297	1.88 × 10^−4^
Nc	0.6	0.90335229	0.903351487	8.89 × 10^−5^
	0.7	0.94676606	0.946765989	7.55 × 10^−6^
	0.8	0.98905069	0.98904967	1.03 × 10^−4^
χ	0.1	0.66797434	0.66796591	1.26 × 10^−3^
	0.2	0.71760717	0.71760249	6.52 × 10^−4^
	0.3	0.7659305	0.76592721	4.30 × 10^−4^
Nr	0.25	0.78643885	0.7864309	1.01 × 10^−3^
	0.35	0.81624387	0.816243085	9.62 × 10^−5^
	0.45	0.84486142	0.844860437	1.16 × 10^−4^

**Table 4:** Validation of the GA-ANN model with previously published work.

Nc	0.25	1.00	2.25	4.00
**Khaled** [64]	0.886819	0.648054	0.425096	0.265802
**ANN**	0.886769	0.648018	0.425041	0.265728
**Error % (ANN)**	0.005638	0.005555	0.012938	0.027840
**GA-ANN**	0.886818	0.648055	0.425097	0.265803
**Error % (GA-ANN)**	0.000112	0.000154	0.000235	0.000376

## Data Availability

The authors confirm that the data supporting the findings of this study are available within the article.

## Nomenclature

$a_{RL}$	Fin profile aspect ratio
A	Area
$b_{1}$	Variable parameter
$c_{p}$	Specific heat
$h_{D}$	Mass transfer convective coefficient
$h^{*}\left(T\right)$	Convective heat transfer coefficient
H	Fin half height
$H_{0}$	Fin base half height
$i_{fg}$	Latent heat of evaporation
$k^{*}\left(T\right)$	Thermal conductivity
L	Fin’s length
Le	Lewis number
m	Exponent constant
n	Wave number
Nc	Convection–conduction parameter
Nr	Radiation–conduction parameter
Q	Heat transfer rate
T	Temperature
w	Width of the fin
x	Fin axial distance
X	Fin’s length (dimensionless)
	Greek symbols
β	Thermal conductivity parameter
δ	Surface wave dimensionless amplitude
$\tilde{\kappa }$	Thermal conductivity coefficient
φ	Surface wave phase shift
ω	Humidity ratio of the saturated air
Θ	Non-dimensional temperature
χ	Wet parameter
	Subscripts
amb	Ambient
b	Base
CS	Cross-section
SF	Surface area
	Abbreviations
ANN	Artificial neural network
GA	Genetic algorithm
GA-ANN	Genetic algorithm-artificial neural network
GA-ENN	Genetic algorithm-enhanced neural network
ME	Maximum error
MAE	Mean absolute error
MSE	Mean squared error
ODE	Ordinary differential equation
RKF-45	Runge–Kutta–Fehlberg’s fourth–fifth method
SDWF	Surface dry wavy fin
SWWF	Surface wetted wavy fin

## References

[1] McGlen R.J., Jachuck R., Lin S. (2004). Integrated thermal management techniques for high power electronic devices. Appl. Therm. Eng..

[2] Yi L., Hu H., Li C., Zhang Y., Yang S., Pan M. (2021). Experimental investigation on enhanced flow and heat transfer performance of micro-jet impingement vapor chamber for high power electronics. Int. J. Therm. Sci..

[3] Jafari S., Nikolaidis T. (2018). Thermal Management Systems for Civil Aircraft Engines: Review, Challenges and Exploring the Future. Appl. Sci..

[4] Liu H., Wen M., Yang H., Yue Z., Yao M. (2021). A Review of Thermal Management System and Control Strategy for Automotive Engines. J. Energy Eng..

[5] Rahman M.M., Dongxu J., Beni M.S., Hei H.C., He W., Zhao J. (2016). Supercritical water heat transfer for nuclear reactor applications: A review. Ann. Nucl. Energy.

[6] Akay O.E., Das M. (2021). Modeling the total heat transfer coefficient of a nuclear research reactor cooling system by different methods. Case Stud. Therm. Eng..

[7] Huang J., Wang C., Guo K., Zhang D., Su G., Tian W., Qiu S. (2022). Heat transfer analysis of heat pipe cooled device with thermoelectric generator for nuclear power application. Nucl. Eng. Des..

[8] Kim H.J., Yoon H.S. (2018). Forced convection heat transfer from the biomimetic cylinder inspired by a harbor seal vibrissa. Int. J. Heat Mass Transf..

[9] Li G., Wang Z., Wang F., Zhang Y. (2022). Numerical investigation on the performance characteristics of a novel biomimetic honeycomb fractal gas cooler of transcritical CO_2_ heat pump. J. Build. Eng..

[10] Liang H., You S., Zhang H. (2015). Comparison of different heat transfer models for parabolic trough solar collectors. Appl. Energy.

[11] Sandeep H., Arunachala U. (2017). Solar parabolic trough collectors: A review on heat transfer augmentation techniques. Renew. Sustain. Energy Rev..

[12] Said S., Mellouli S., Alqahtani T., Algarni S., Ajjel R. (2023). New Evacuated Tube Solar Collector with Parabolic Trough Collector and Helical Coil Heat Exchanger for Usage in Domestic Water Heating. Sustainability.

[13] Appadurai M., Raj E.F.I., Ram V., Gnaniah A.M., Salkuti S.R., Kim S.-C. (2023). Investigation of Solar Air Collectors with Carbon-Nanotube-Based Turbulators and Pin Fin Arrangements. J. Compos. Sci..

[14] Said S., Mellouli S., Alqahtani T., Algarni S., Ajjel R., Ghachem K., Kolsi L. (2023). An Experimental Comparison of the Performance of Various Evacuated Tube Solar Collector Designs. Sustainability.

[15] Patil M.S., Seo J.-H., Kang S.-J., Lee M.-Y. (2016). Review on Synthesis, Thermo-Physical Property, and Heat Transfer Mechanism of Nanofluids. Energies.

[16] Yu X., Wu Q., Zhang H., Zeng G., Li W., Qian Y., Yang G., Chen M. (2018). Investigation on Synthesis, Stability, and Thermal Conductivity Properties of Water-Based SnO2/Reduced Graphene Oxide Nanofluids. Materials.

[17] Awais M., Bhuiyan A.A., Salehin S., Ehsan M.M., Khan B., Rahman M.H. (2021). Synthesis, heat transport mechanisms and thermophysical properties of nanofluids: A critical overview. Int. J. Thermofluids.

[18] Rashidi M.M., Nazari M.A., Mahariq I., El Haj Assad M., Ali M.E., Almuzaiqer R., Nuhait A., Murshid N. (2021). Thermophysical Properties of Hybrid Nanofluids and the Proposed Models: An Updated Comprehensive Study. Nanomaterials.

[19] Lu D., Ramzan M., Mohammad M., Howari F., Chung J.D. (2019). A Thin Film Flow of Nanofluid Comprising Carbon Nanotubes Influenced by Cattaneo-Christov Heat Flux and Entropy Generation. Coatings.

[20] Turkyilmazoglu M. (2019). Fully developed slip flow in a concentric annuli via single and dual phase nanofluids models. Comput. Methods Programs Biomed..

[21] Suleman M., Ramzan M., Ahmad S., Lu D., Muhammad T., Chung J.D. (2019). A Numerical Simulation of Silver–Water Nanofluid Flow with Impacts of Newtonian Heating and Homogeneous–Heterogeneous Reactions Past a Nonlinear Stretched Cylinder. Symmetry.

[22] Haq I., Yassen M.F., Ghoneim M.E., Bilal M., Ali A., Weera W. (2022). Computational Study of MHD Darcy–Forchheimer Hybrid Nanofluid Flow under the Influence of Chemical Reaction and Activation Energy over a Stretching Surface. Symmetry.

[23] Ramzan M., Riasat S., Aljurbua S.F., Ghazwani H.A.S., Mahmoud O. (2022). Hybrid Nanofluid Flow Induced by an Oscillating Disk Considering Surface Catalyzed Reaction and Nanoparticles Shape Factor. Nanomaterials.

[24] Alsulami M.D., Abdulrahman A., Kumar R.N., Gowda R.J.P., Prasannakumara B.C. (2023). Three-Dimensional Swirling Flow of Nanofluid with Nanoparticle Aggregation Kinematics Using Modified Krieger–Dougherty and Maxwell–Bruggeman Models: A Finite Element Solution. Mathematics.

[25] Alharbi K.A.M., Bilal M., Ali A., Eldin S.M., Alburaikan A., Khalifa H.A.E.-W. (2023). Significance of gyrotactic microorganisms on the MHD tangent hyperbolic nanofluid flow across an elastic slender surface: Numerical analysis. Nanotechnol. Rev..

[26] Nagaraja K.V., Khan U., Madhukesh J.K., Hassan A.M., Prasannakumara B.C., Kahla N.B., Elattar S., Chohan J.S. (2023). Heat and mass transfer analysis of assisting and opposing radiative flow conveying ternary hybrid nanofluid over an exponentially stretching surface. Sci. Rep..

[27] Adnan, Nadeem A., Mahmoud H.A., Ali A., Eldin S.M. (2023). Significance of Koo-Kleinstreuer-Li model for thermal enhancement in nanofluid under magnetic field and thermal radiation factors using LSM. Adv. Mech. Eng..

[28] Illán F., Alarcón M. (2011). Optimization of Annular Cylindrical and Spherical Fins in an Internal Combustion Engine Under Realistic Conditions. J. Therm. Sci. Eng. Appl..

[29] Kang H.C., Jun G.W. (2011). Heat Transfer and Flow Resistance Characteristics of Louver Fin Geometry for Automobile Applications. J. Heat Transf..

[30] Shinde A., Arpit S., KM P., Rao P.V.C., Saha S.K. (2017). Heat Transfer Characterization and Optimization of Latent Heat Thermal Storage System Using Fins for Medium Temperature Solar Applications. J. Sol. Energy Eng..

[31] Alnaimat F., Ziauddin M. (2021). Experimental investigation of heat transfer in pin-fins heat sinks for cooling applications. Heat Mass Transf..

[32] Jiang Q., Pan C., Chen Y., Zhang Q., Tang Y., Gu J., Aleksandr P. (2021). Improved heat transfer and friction correlations of aluminum offset-strip fin heat exchangers for helium cryogenic applications. Appl. Therm. Eng..

[33] Kundu B., Lee K.-S. (2012). Analytic solution for heat transfer of wet fins on account of all nonlinearity effects. Energy.

[34] Turkyilmazoglu M. (2014). Efficiency of heat and mass transfer in fully wet porous fins: Exponential fins versus straight fins. Int. J. Refrig..

[35] Das R., Kundu B. (2018). Direct and inverse approaches for analysis and optimization of fins under sensible and latent heat load. Int. J. Heat Mass Transf..

[36] Hazarika S.A., Bhanja D., Nath S. (2020). Fork-shaped constructal fin array design a better alternative for heat and mass transfer augmentation under dry, partially wet and fully wet conditions. Int. J. Therm. Sci..

[37] Gamaoun F., Said N.M., Makki R., Kumar R.S.V., Sowmya G., Prasannakumara B.C., Kumar R. (2022). Energy transfer of a fin wetted with ZnO-SAE 50 nanolubricant:An application of α-parameterized differential transform method. Case Stud. Therm. Eng..

[38] Abdulrahman A., Gamaoun F., Kumar R.S.V., Khan U., Gill H.S., Nagaraja K.V., Eldin S.M., Galal A.M. (2023). Study of thermal variation in a longitudinal exponential porous fin wetted with TiO2−SiO2/hexanol hybrid nanofluid using hybrid residual power series method. Case Stud. Therm. Eng..

[39] Xiao L., Wu T., Feng S., Du X., Yang L. (2017). Experimental study on heat transfer enhancement of wavy finned flat tubes by water spray cooling. Int. J. Heat Mass Transf..

[40] Wen J., Li K., Wang C., Zhang X., Wang S. (2019). Optimization investigation on configuration parameters of sine wavy fin in plate-fin heat exchanger based on fluid structure interaction analysis. Int. J. Heat Mass Transf..

[41] Chu W.-X., Sheu W.-J., Hsu C.-C., Wang C.-C. (2020). Airside performance of sinusoidal wavy fin-and-tube heat exchangers subject to large-diameter tubes with round or oval configuration. Appl. Therm. Eng..

[42] Zhang K., Li M.-J., Liu H., Xiong J.-G., He Y.-L. (2021). Experimental and numerical study and comparison of performance for herringbone wavy fin and enhanced fin with convex-strips in fin-and-tube heat exchanger. Int. J. Heat Mass Transf..

[43] Erdinc M.T. (2023). Computational thermal-hydraulic analysis and geometric optimization of elliptic and circular wavy fin and tube heat exchangers. Int. Commun. Heat Mass Transf..

[44] Kumar V., Chhabra J.K., Kumar D. (2014). Parameter adaptive harmony search algorithm for unimodal and multimodal optimization problems. J. Comput. Sci..

[45] Michalewicz Z., Schoenauer M. (1996). Evolutionary Algorithms for Constrained Parameter Optimization Problems. Evol. Comput..

[46] Le L.T., Nguyen H., Dou J., Zhou J. (2019). A Comparative Study of PSO-ANN, GA-ANN, ICA-ANN, and ABC-ANN in Estimating the Heating Load of Buildings’ Energy Efficiency for Smart City Planning. Appl. Sci..

[47] Albadr M.A., Tiun S., Ayob M., AL-Dhief F. (2020). Genetic Algorithm Based on Natural Selection Theory for Optimization Problems. Symmetry.

[48] Zhang Y., Pan G., Chen B., Han J., Zhao Y., Zhang C. (2020). Short-term wind speed prediction model based on GA-ANN improved by VMD. Renew. Energy.

[49] Hamdia K.M., Zhuang X., Rabczuk T. (2021). An efficient optimization approach for designing machine learning models based on genetic algorithm. Neural Comput. Appl..

[50] Let S., Bar N., Basu R.K., Das S.K. (2023). Bed expansion of binary mixtures of irregular particles in solid–liquid fluidization: Experimental, empirical correlation, and GA-ANN modelling. Can. J. Chem. Eng..

[51] Sharifi K., Sabeti M., Rafiei M., Mohammadi A.H., Ghaffari A., Asl M.H., Yousefi H. (2020). A good contribution of computational fluid dynamics (CFD) and GA-ANN methods to find the best type of helical wire inserted tube in heat exchangers. Int. J. Therm. Sci..

[52] Wen T., Zhu G., Jiao K., Lu L. (2021). Experimental study on the thermal and flow characteristics of ZnO/water nanofluid in mini-channels integrated with GA-optimized ANN prediction and CFD simulation. Int. J. Heat Mass Transf..

[53] Cui W., Si T., Li X., Li X., Lu L., Ma T., Wang Q. (2022). Heat transfer analysis of phase change material composited with metal foam-fin hybrid structure in inclination container by numerical simulation and artificial neural network. Energy Rep..

[54] Hosseinzadeh S., Hosseinzadeh K., Hasibi A., Ganji D.D. (2022). Thermal analysis of moving porous fin wetted by hybrid nanofluid with trapezoidal, concave parabolic and convex cross sections. Case Stud. Therm. Eng..

[55] Gouran S., Ghasemi S.E., Mohsenian S. (2022). Effect of internal heat source and non-independent thermal properties on a convective–radiative longitudinal fin. Alex. Eng. J..

[56] Ullah I., Ullah S., Ali A., Shah S.I., Weera W., Alam M.M. (2022). Heat transfer analysis from moving convection-radiative triangular porous fin exposed to heat generation. Case Stud. Therm. Eng..

[57] Adnan, AlBaidani M.M., Mishra N.K., Alam M.M., Eldin S.M., AL-Zahrani A.A., Akgul A. (2023). Numerical analysis of magneto-radiated annular fin natural-convective heat transfer performance using advanced ternary nanofluid considering shape factors with heating source. Case Stud. Therm. Eng..

[58] Gireesha B.J., Keerthi M.L., Eshwarappa K.M. (2023). Transient thermal investigation of a fully wet porous convective–radiative rough cylindrical pin fin. Heat Transf..

[59] Hashemi A.S., Heydari M., Loghmani G.B. (2023). Iterative compact finite difference method for the numerical study of fully wet porous fins with different profile shapes. Appl. Numer. Math..

[60] Kumar R.S.V., Alsulami M.D., Sarris I.E., Sowmya G., Gamaoun F. (2023). Stochastic Levenberg–Marquardt Neural Network Implementation for Analyzing the Convective Heat Transfer in a Wavy Fin. Mathematics.

[61] Kundu B., Yook S.-J. (2023). Analytical model for extremum analysis of moistened fins involving all nonlinear energy exchange processes. Case Stud. Therm. Eng..

[62] Kumar C., Nimmy P., Nagaraja K.V., Kumar R.S.V., Verma A., Alkarni S., Shah N.A. (2023). Analysis of Heat Transfer Behavior of Porous Wavy Fin with Radiation and Convection by Using a Machine Learning Technique. Symmetry.

[63] Din Z.U., Ali A., Khan Z.A., Zaman G. (2023). Investigation of moving trapezoidal and exponential fins with multiple nonlinearities. Ain Shams Eng. J..

[64] Khaled A.A. (2015). Thermal performance of six different types of wavy-fins. Int. J. Numer. Methods Heat Fluid Flow.

